# Editorial: Harnessing crop biodiversity and genomics assisted pre-breeding approaches for next generation climate-smart varieties, volume II

**DOI:** 10.3389/fpls.2024.1387016

**Published:** 2024-03-06

**Authors:** Prashant Vikram, Deepmala Sehgal, Manoj Prasad, Sukhwinder Singh, Harsh Raman

**Affiliations:** ^1^ Director Research, Faculty of Agricultural Sciences, Shree Gurugobind Singh Tricentenary University, Gurugram, Haryana, India; ^2^ Research and Development, Syngenta, Jealott’s Hill International Research Centre, Bracknell, United Kingdom; ^3^ Department of Genetics, University of Delhi, New Delhi, India; ^4^ United States Department of Agriculture, Agricultural Research Service, Subtropical Horticulture Research Station, Miami, FL, United States; ^5^ NSW Department of Primary Industries, Agriculture, Wagga Wagga Agricultural Institute PMB, Wagga Wagga, NSW, Australia

**Keywords:** germplasm accession, pre breeding, genomics assisted breeding, genebank, crops, landraces

Agricultural innovation is paramount to broadening the genetic diversity of crops, focusing on enhancing yield, tolerance to biotic and abiotic stress factors nutritional value and adaptation to new environments, especially in response to climate change. Leveraging diverse genetic resources, including on-farm diversity and germplasm maintained in Gene Banks including local landraces, and secondary, and tertiary gene pools has become imperative. Traditional varieties, landraces and other under-utilized germplasms are seldom used by breeders mainly due to the unwanted linkages. Genomics tools can help in handling this efficiently. For instance, the “genetic linkage between *sd1* gene and drought tolerance QTL” in rice is a significant breeding challenge that has recently been overcome through marker-assisted breeding. Another example is the “CIMMYT - Seeds of discovery (SeeD)” initiative, which used genomics tools to enable the large-scale usage of wheat germplasm banks. Advanced genomics tools and technologies offer promising avenues for varietal development through knowledge enrichment which is instrumental in strategizing breeding programs. By integrating underutilized and unlocking genetic diversity by identifying and incorporating novel alleles, the genetic base of cultivated varieties can be broadened. This approach, termed “genomics-assisted-pre-breeding,” encompasses diversity analysis, functional genomics, and structural genomics, in combination with advanced statistical tools necessary for crop improvement. Embracing “genomics-assisted-pre-breeding” is critical for breeders worldwide to meet global food, fuel and fibre demands. Moving beyond the Green Revolution means looking towards innovative strategies that harness the full potential of crop biodiversity to meet future food demands.

Wheat is one of the most widely grown crops in the world and unveiling its genetic diversity is important for genetic improvement programs. Ali et al. investigated a set of 422 wheat accessions including synthetic derivatives, cultivars and breeding lines for nucleotide diversity, population structure, and selection signatures in a breeding program?. The study identified 32 unique genome regions which were subjected selection pressure. Among these regions, B, D & A genomes contribute 50%, 29% and 21% respectively. Interestingly, these regions harboured genes/QTLs controlling adaptive traits including vernalization, adaptability, disease resistance, and yield-components.

Miazzi et al. investigated the relationships between the Tunisian durum landraces (*Triticum turgidum* L. ssp. durum Desf.) and the modern cultivars. This study identified candidate genes such as transcription factors AP2/EREBPs, zinc finger CONSTANS, and FLOWERING LOCUS T (FT-B1) for plant and spike architecture. Furthermore, distinct genes related to grain composition, disease resistance proteins (NPS-LRR and RPM), and nucleotide-binding site and leucine-reach repeat proteins in response to biotic stress were identified.

In another study, Mulugeta et al. assessed genetic diversity, population structure, and linkage disequilibrium in five hundred (500) lines including landraces, cultivars and breeding lines from China and, identified regions under selection. With 65 loci under balancing selection and 17 under directional selection, the genomic scan employing the Fst outlier test identified 85 selection signatures. Potential candidate genes were linked to grain yield, plant height, host plant susceptibility to diseases, heading date, grain quality, and phenolic content when they co-localized with genomic regions showing significant selection signals. The genotypes were grouped into five subpopulations, with clustered landraces from geographically non-adjoining environments.

In addition to broadening the genetic base, landraces also serve as a genetic repertoire for abiotic stress tolerance breeding programs including drought, heat, salinity etc. Barratt et al. analysed a landrace panel (YoGI panel) to identify genes that express differentially under early-stage drought stress situations and unveiled two novel hub candidate genes – one as an activator (TaDHN4-D1; TraesCS5D02G379200) and the other as a repressor (uncharacterised gene; TraesCS3D02G361500). Further, Barratt et al. determined that two promising candidate hub genes (TraesCS3B02G409300 and TraesCS1B02G384900) may downregulate the expression of genes involved in drought, salinity, and cold responses—genes that are unlikely to be required under heat stress—as well as photosynthesis genes and stress hormone signalling repressors. Salami et al. studied metabolic profiles of 119 rapeseed (*Brassica napus*) accessions under drought stress conditions, which prioritised 60 candidate genes, of which 18 were transcription factors were involved in the stress-induced pathways, phenylpropanoid pathway and flavonoid modifications. Among these candidate genes, PAL1, CHI, UGT89B1, FLS3, CCR1, and CYP75B137 contributed to flavonoid biosynthetic pathways.

These studies revealed that germplasm collections are rich reservoir of genetic diversity which can be harnessed to develop next generation climate smart varieties etc Genomics tools and approaches will continue to play a critical role in enhancing breeding efficiency. Specifically, germplasm resources (landraces, wild relatives etc.) can be subjected to a variety of genomic analyses for knowledge enrichment and for practical utilization in breeding programs. For example, core set formulation, genetic relatedness, candidate gene information etc are quite relevant for strategizing breeding programs. Genetic and genomic knowledge generated by diverse germplasm accessions including genetic relatedness and stress-related functional candidate genes should help greatly in modernizing current and future breeding programs.

## Author contributions

PV: Writing – original draft, Writing – review & editing. DS: Writing – original draft, Writing – review & editing. MP: Writing – original draft, Writing – review & editing. SS: Writing – original draft, Writing – review & editing. HR: Writing – original draft, Writing – review & editing.

